# Medication as a risk factor for hospitalization due to heart failure and shock: a series of case-crossover studies in Swiss claims data

**DOI:** 10.1007/s00228-020-02835-x

**Published:** 2020-04-08

**Authors:** Annika M. Jödicke, Andrea M. Burden, Urs Zellweger, Ivan T. Tomka, Thomas Neuer, Malgorzata Roos, Gerd A. Kullak-Ublick, Ivanka Curkovic, Marco Egbring

**Affiliations:** 1 Department of Clinical Pharmacology and Toxicology, University Hospital Zurich, University of Zurich, Zurich, Switzerland; 2 Institute of Pharmaceutical Sciences, Swiss Federal Institute of Technology Zurich (ETH Zurich), Zurich, Switzerland; 3 Institute of Pharmaceutical Sciences, Division of Pharmacoepidemiology, Swiss Federal Institute of Technology Zurich (ETH Zurich), Zurich, Switzerland; 4 Department of Client Services & Claims, Helsana Group, Zurich, Switzerland; 5 EPha.ch AG, Project Drug Safety, Zurich, Switzerland; 6 EBPI, Department of Biostatistics, University of Zurich, Zurich, Switzerland

**Keywords:** Pharmacoepidemiology, Antibiotics, Analgesics, Heart failure, Drug safety

## Abstract

**Purpose:**

Heart failure is among the leading causes for hospitalization in Europe. In this study, we evaluate potential precipitating factors for hospitalization for heart failure and shock.

**Methods:**

Using Swiss claims data (2014–2015), we evaluated the association between hospitalization for heart failure and shock, and prescription of oral potassium supplements, non-steroidal anti-inflammatory drugs (NSAIDs), and amoxicillin/clavulanic acid. We conducted case-crossover analyses, where exposure was compared for the hazard period and the primary control period (e.g., 1–30 days before hospitalization vs. 31–60 days, respectively). Conditional logistic regression was applied and subsequently adjusted for addressing potential confounding by disease progression. Sensitivity analyses were conducted and stratification for co-medication was performed.

**Results:**

We identified 2185 patients hospitalized with heart failure or shock. Prescription of potassium supplements, NSAIDs, and amoxicillin/clavulanic acid was significantly associated with an increased risk for hospitalization for heart failure and shock with crude odds ratios (OR) of 2.04 for potassium (95% CI 1.24–3.36, *p* = 0.005, 30 days), OR 1.8 for NSAIDs (95% CI 1.39–2.33, *p* < 0.0001, 30 days), and OR 3.25 for amoxicillin/clavulanic acid (95% CI 2.06–5.14, *p* < 0.0001, 15 days), respectively. Adjustment attenuated odds ratios, while the significant positive association remained (potassium OR 1.70 (95% CI 1.01–2.86, *p* = 0.046), NSAIDs OR 1.50 (95% CI 1.14–1.97, *p* = 0.003), and amoxicillin/clavulanic acid OR 2.26 (95% CI 1.41–3.62, *p* = 0.001).

**Conclusion:**

Prescription of potassium supplements, NSAIDs, and amoxicillin/clavulanic acid is associated with increased risk for hospitalization. Underlying conditions such as pain, electrolyte imbalances, and infections are likely contributing risk factors. Physicians may use this knowledge to better identify patients at risk and adapt patient management.

**Electronic supplementary material:**

The online version of this article (10.1007/s00228-020-02835-x) contains supplementary material, which is available to authorized users.

## Introduction

Heart failure (HF) is among the leading causes for hospitalization in Europe, especially in the elderly [1], with an estimated prevalence of 1–2% in the western world [2]. Gaining insight into the development of patients’ healthcare-seeking behavior towards hospitalization and subsequent identification of risk factors for HF decompensation is of great importance for improved risk stratification and management [3].

The American Heart Association released a list of medications that could exacerbate HF [4], covering drugs from thirteen distinct therapeutic classes. Unfortunately, prescriptions for these medications in HF patients are still common. A study evaluating National Health and Nutrition Examination Survey data from the USA found a prevalence of 48% of heart failure exacerbating medication use [5].

Examples for common drug mechanisms that may favor HF deterioration comprise direct myocardial toxicity and negative inotropic or chronotropic effects, but drugs may also exacerbate hypertension, deliver high sodium loads, or cause drug-drug interactions that may limit the beneficial effects of (other) HF medications [4]. Several studies have assessed the association between prescriptions of non-steroidal anti-inflammatory drugs (NSAIDs) and HF hospitalization [6–11]. The hypothesized underlying mechanism is the inhibition of the prostaglandin synthesis, potentially resulting in reduced renal perfusion, glomerular filtration rate, and increased peripheral systemic resistance [7].

While medications themselves may be risk factors for decompensation or development of HF, they can also serve as proxies for acute conditions presenting potential risk factors. Conditions such as arrhythmia, worsening renal function, and infections [12, 13] are common complications in patients suffering from HF and were described to be precipitating factors for HF hospitalization [3, 14–18]. Additionally, electrolyte imbalances appear frequently in HF patients [19–22], especially as several drugs commonly used in heart failure treatment may lead to hypo- or hyperkalemia, respectively [23, 24]. Although prescription of potassium supplements was previously described to be associated with HF hospitalization in the long-term [22], and “infection” is reported to be a common precipitating factor, neither prescriptions of potassium nor amoxicillin/clavulanic acid have been assessed as acute risk factors for heart failure hospitalization.

### Objective

The objective of this study was to evaluate the prescription of potassium supplements, NSAIDs, and amoxicillin/clavulanic acid as precipitating factors for hospitalization for heart failure and shock. The development of patients’ healthcare-seeking behavior over time towards hospitalization was assessed and used to address potential confounding for disease progression.

## Methods

### Claims data

We conducted three case-crossover analyses using anonymized health insurance data on Swiss adults. The dataset was provided by the Helsana Group, which is one of the largest health insurance companies in Switzerland covering approximately 1.2 million individuals (compulsory health insurance) [25]. While health insurance is mandatory for all residents of Switzerland, residents are free to choose their preferred insurance provider. The recorded insurance claims cover all healthcare invoices submitted to the Helsana Group for reimbursement [25]. We obtained data from the years 2014–2015.

Our dataset included patient demographics and records of utilized healthcare including claimed drug prescriptions, hospitalizations, and physician visits. Patients were required to have at least five drug prescriptions within a calendar year and no private additional health insurance. Hospitalization was identified using Swiss Diagnosis Related Groups (SwissDRGs) [26], which is used for the invoicing of inpatient hospitalization services. Based on specific criteria such as the primary and secondary diagnoses, treatments, and degree of severity [27], each hospitalization is assigned to a SwissDRG. Due to the legal requirements in Switzerland, no explicit diagnoses were accessible in health insurance data. Outpatient physician visits and diagnostic procedures performed in the outpatient setting were identified using TARMED codes [28].

### Study design

**Fig. 1:**
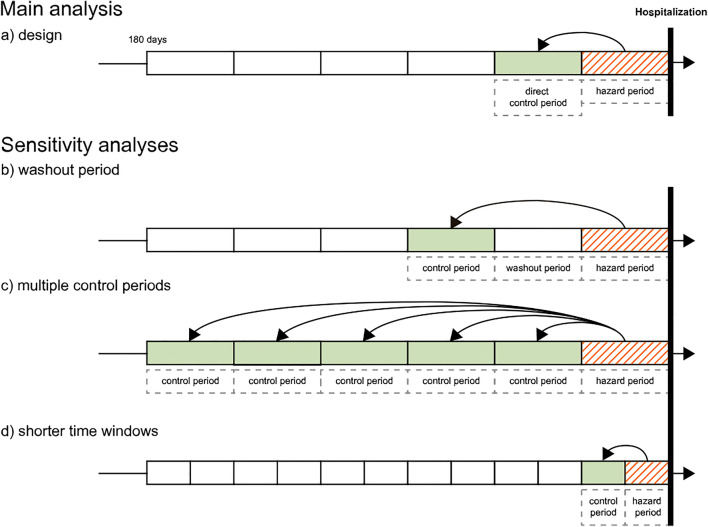
Case-crossover design. As a case-only design, patients serve both as cases and their own controls. Main analysis: For each patient, exposure in the “hazard” period immediately preceding the outcome was compared with exposure in the “direct control” period earlier in time (**a**). Hazard and control period were equal in length. Sensitivity analyses: Introduction of a single washout period to prevent exposure overlapping from the control into the subsequent hazard period (**b**). Inclusion of multiple control periods simultaneously to assess the impact of more distant periods and to account for persistent drug use (**c**). Finally, we evaluated shorter time periods for hazard and control periods (**d**)

Three independent case-crossover studies were conducted to study oral potassium supplements, NSAIDs, and amoxicillin/clavulanic acid as potential risk factors for hospitalization. The case-crossover design is ideal to assess associations between transient risk factors, such as short-term medication utilization, and outcomes with abrupt onset [29], such as hospitalization for heart failure or shock. The design controls for time-invariant covariates [29, 30], thereby minimizing the potential for confounding due to stable (measurable and immeasurable) risk factors [9]. As a case-only design, patients serve both as cases and their own controls. Since exposure to potassium supplements, NSAIDs, and antibiotics is considered to be a transient effect, each patient can be “exposed” and “not exposed” at different time periods. For each patient, exposure in the “hazard” period immediately preceding the outcome was compared with exposure in “control” period(s) earlier in time [29, 31]. The main study design is displayed in Fig. 1 a.

Sensitivity analyses included introducing a single washout period to prevent exposure overlapping from the control into the subsequent hazard period, using multiple control periods simultaneously, thereby minimizing the effect of confounding by disease progression or exposure misclassification, and shortening of the time periods (Fig. 1b–d). Additionally, we excluded patients with all-cause hospitalizations within the 180-day observation period, as previous studies found recent hospitalizations to be an independent risk factor for hospitalization for heart failure [6].

### Case selection

All patients with hospitalizations for heart failure and shock were identified as cases using SwissDRG codes (F62A, F62B, and F62C, Supplement Table S1) [26]. For each case, the first hospitalization within the study period was defined as index hospitalization. An observation period of at least 180 days prior to the index hospitalization was required, and patients were excluded if they had a nursing home stay during that period. The inclusion process is displayed in Fig. S1 in the supplement in detail.

### Exposure definition

Drug prescriptions were identified using 5th-level Anatomical Therapeutic Chemical (ATC) code [32]. The primary exposures of interest were oral potassium supplements (ATC: A12BA), NSAIDs (ATC: M01AA, M01AB, M01AC, M01AE, M01AG, M01AH, M01AX12), and amoxicillin/clavulanic acid (ATC: J01CR02). Once the respective drug was prescribed at any time within the hazard or control period, the patient was considered to be “exposed” for that period.

### Statistical analysis

Data were analyzed using R software (version 3.4.0). For each patient, demographic parameters, claimed drug prescriptions, and utilized healthcare such as hospitalizations, physician visits, and diagnostics/laboratory procedures were analyzed within the 180-day observation period prior to index hospitalization. For dichotomous variables, absolute and relative frequencies were calculated. For continuous variables, medians and interquartile range (IQR) were provided. For trend analysis, we visualized the frequency of daily outpatient physician visits towards index hospitalization. To reduce short-term variations, visits were binned into 7-day intervals. Based on the binned visits, a Spearman correlation coefficient was calculated.

For the case-crossover study, we identified discordant pairs and calculated Mantel-Haenszel odds ratio estimates (OR, with 95% confidence intervals) using conditional logistic regression to compare the odds of exposure to one of the drugs of interest in the hazard period to the exposure in the control periods. Adjusted ORs were calculated to control for the potential of time-variant confounding resulting from disease progression and deterioration of the ejection fraction. We concurrently adjusted for the number of outpatient physician visits per time period and the co-prescription of high-ceiling diuretics (sulfonamides, C03CA).

The hazard and control periods were defined according to the exposure of interest as follows: Potassium: 30 days (to cover acute hypokalemia and account for possible potassium accumulation); NSAIDs: 30 days (in accordance with previous studies [6, 8, 9, 11, 33]); and amoxicillin/clavulanic acid: 15 days (to allow for treatment durations of usually 1–2 weeks depending on indication and severity). Shortened time periods were used as sensitivity analyses (Fig. 1d): Potassium and NSAIDs (20- or 14-day periods) and amoxicillin/clavulanic acid (10- or 7-day periods).

### Additional analyses

The acute conditions of “pain” and “bacterial infection” serve as the indication for NSAIDs and amoxicillin/clavulanic acid. As these conditions may present risk factors on their own, we subsequently conducted additional case-crossover studies: To evaluate the overall indication of “pain,” we independently evaluated paracetamol, metamizole sodium, and opioids as drugs defining “exposure” instead of NSAIDs. Accordingly, we evaluated “bacterial infection” as a risk factor and conducted a case-crossover study for patients with other antibiotic classes.

Finally, we stratified the analyses to examine the role of interacting co-medications. For potassium supplements, we evaluated potassium-sparing and potassium-depleting co-medication (ace-inhibitors/sartans, high-ceiling diuretics (sulfonamides)) [34]; for NSAIDs, co-medication with diuretics, ace-inhibitors/sartans, anticoagulants, and platelet inhibitors was evaluated. For amoxicillin/clavulanic acid, we repeated the primary analysis in patients suffering from asthma or chronic obstructive pulmonary disease (COPD).

## Results

### Patient characteristics and healthcare utilization

A total of 2185 patients met our inclusion criteria. A summary of the patients’ characteristics is provided in Table 1. Patients were old and had a median age over 80 years. Approximately one-third of the patients were previously hospitalized for other causes, highlighting disease burden. A substantial amount of them experienced other cardiac-related hospitalizations.

**Table 1:** Demographics and healthcare utilization

		All patients	Potassium supplement	NSAIDs	Amoxicillin/clavulanic acid
*N*		2185	118	543	318
DRG “Heart failure and shock” (*N*, %)					
With severe co-morbidities (F62A, F62B)		572 (26.2%)	37 (31.4%)	130 (23.9%)	103 (32.4%)
Without severe co-morbidities (F62C)		1613 (73.8%)	81 (68.6%)	413 (76.1%)	215 (67.6%)
Age (Median, IQR)		82.6 (75.9, 87.9)	81.3 (74.2, 86.5)	82.0 (75.9, 87.2)	81.5 (73.6, 87.2)
Gender (F) (*N*, %)		1092 (50.0%)	65 (55.1%)	296 (54.5%)	124 (39.0%)
Previous hospitalizations (*N*, %)					
All other causes					
30 days		267 (12.2%)	19 (16.1%)	82 (15.1%)	44 (13.8%)
180 days		741 (33.9%)	58 (49.2%)	196 (36.1%)	149 (46.9%)
Cardiac-related^a^					
30 days		56 (2.6%)	9 (7.6%)	16 (2.9%)	6 (1.9%)
180 days		259 (11.9%)	23 (19.5%)	58 (10.7%)	46 (14.5%)
Number of visits (Median, IQR)					
30 days		2 (1, 3)	3 (1, 4)	2 (1, 3)	2 (1, 4)
180 days		8 (4, 12)	10 (7, 17)	9 (5, 13)	10 (7, 14)
Number of drugs (Median, IQR)					
180 days		13 (9, 17)	16 (13, 23)	15 (11, 20)	16 (11, 14)
Laboratory assessment^b^ (*N*, %)					
BNP	30 days	245 (11.2%)	19 (16.1%)	73 (13.4%)	34 (10.7%)
	180 days	489 (22.4%)	39 (33.1%)	125 (23.0%)	74 (23.3%)
GFR	30 days	767 (35.1%)	71 (60.2%)	210 (38.7%)	149 (46.9%)
	180 days	1539 (70.4%)	104 (88.1%)	413 (76.1%)	255 (80.2%)
Potassium	30 days	628 (28.7%)	72 (61.0%)	161 (29.7%)	118 (37.1%)
	180 days	1315 (60.2%)	107 (90.7%)	339 (62.4%)	219 (68.9%)
Diagnostic procedures^c^ (*N*, %)					
Electrocardiography	30 days	396 (18.1%)	28 (23.7%)	121 (22.3%)	75 (23.6%)
	180 days	855 (39.1%)	53 (44.9%)	234 (43.1%)	147 (46.2%)
Echocardiography	30 days	136 (6.2%)	11 (9.3%)	37 (6.8%)	18 (5.7%)
	180 days	390 (17.8%)	28 (23.7%)	100 (18.4%)	62 (19.5%)

F62A: Heart failure or shock with severe comorbidities and dialysis, reanimation, or additionally complicating diagnosis; F62B: Heart failure or shock with severe comorbidities but without dialysis, reanimation, or additionally complicating diagnosis; F62C: Heart failure or shock without severe comorbidities; *IQR*, interquartile range; *BNP*, brain natriuretic peptide; *GFR*, glomerular filtration rate

^a^Previous cardiac-related hospitalizations were derived from the major diagnostic categories (SwissDRG)

^b^Number of patients with at least one measure of the respective test within 30 days or 180 days prior to hospital admission. No laboratory values were available

^c^Only tests performed in the outpatient setting were assessed

#### Outpatient physician visits

Most patients (95.3%) had at least one physician office visit within 180 days prior to index hospitalization. Most visits were performed by general practitioners or specialists for internal medicine (63.6%), followed by ophthalmologists, outpatient hospital departments, and cardiologists. With respect to the frequency of outpatient visits, a significant trend for increase towards index hospitalization was found (Spearman *ρ* = 0.56, *p* = 0.0499, Supplement Fig. S2). A total of 677 patients (31%) had an outpatient visit at the day of index hospitalization.

#### Drug prescriptions

Most frequent co-medication were high-ceiling diuretics (sulfonamides), with 31.2% and 62.4% of patients receiving at least one filled prescription within 30 days and 180 days, respectively. Beta-blocking agents, proton-pump inhibitors, platelet inhibitors, and paracetamol were among the 5 most frequently prescribed drug classes both within 30 days and 180 days (Supplement Table S2). Within 30 days prior to index hospitalization, 1403 patients (64.2%) received a prescription of a new drug class. At least one new cardiac therapy was prescribed in 555 patients (39.6%) (ATC starting with “C”). Most frequent *new* drug classes prescribed were high-ceiling diuretics (sulfonamides) (*n* = 184), beta-blocking agents (*n* = 130), paracetamol (*n* = 129), proton pump inhibitors (*n* = 112), and pyrazolones (*n* = 96) (Supplement Table S3). Eighty-eight patients received a new prescription for amoxicillin/clavulanic acid. As previously mentioned, a high number of patients received NSAIDs (*n* = 104) and several patients were prescribed potassium supplements (*n* = 33).

### Case-crossover study

Prescription of potassium supplements and non-steroidal anti-inflammatory drugs was significantly associated with an increased probability for hospitalization for heart failure and shock with odds ratios of 2.04 for potassium (95% CI 1.24–3.36, *p* = 0.005, 30 days) and 1.8 for NSAIDs (95% CI 1.39–2.33, *p* < 0.0001, 30 days) in all 2185 patients. The strongest association was found for amoxicillin/clavulanic acid, with an odds ratio of 3.25 (95% CI 2.06–5.14, *p* < 0.0001, 15 days) (Table 2). Sensitivity analyses introducing a single washout period and the use of multiple control windows confirmed this positive association. Odds ratios for shorter time periods are displayed in Table S4 in the supplement.

**Table 2:** Case-crossover studies

	Windows(days)		Crude Effect	Adj. analysis^a^	*N*	Discordant pairs
	Hazard period	Control period	OR_MH_ (95% CI)	OR_MH_ (95% CI)	With drug (all patients)	Hazard; control
Potassium						
Main analysis	1–30	31–60	2.04 (1.24–3.36) **	1.70 (1.01–2.86) *	118 (2185)	47; 23
Washout	1–30	61–90	5.00 (2.54–9.86) ***	3.60 (1.78–7.26) ***	118 (2185)	50; 10
Multiple control windows	1–30	31–60, 61–90, […], 151–180	3.54 (2.42–5.18) ***	2.56 (1.73–3.79) ***	118 (2185)	CLR^b^
NSAIDs						
Main analysis	1–30	31–60	1.80 (1.39–2.33) ***	1.50 (1.14–1.97) **	543 (2185)	160; 89
Washout	1–30	61–90	1.85 (1.41–2.41) ***	1.64 (1.24–2.18) ***	543 (2185)	155; 84
Multiple control windows	1–30	31–60, 61–90, […], 151–180	1.60 (1.32–1.93) ***	1.29 (1.06–1.56) *	543 (2185)	CLR^b^
Amoxicillin/clavulanic acid						
Main analysis	1–15	16–30	3.25 (2.06–5.14) ***	2.26 (1.41–3.62) ***	318 (2185)	78; 24
Washout	1–15	31–45	3.86 (2.39–6.23) ***	2.41 (1.46–3.97) ***	318 (2185)	81; 21
Multiple control windows	1–15	16–30, 31–45, […], 166–180	3.22 (2.50–4.15) ***	1.87 (1.43–2.44) ***	318 (2185)	CLR^b^

*OR*_*MH*_, Mantel-Haenszel odds ratio; *95% CI*, 95% confidence interval for OR_MH_; *Discordant pairs*, patients with medication exposure in hazard period but not in control period or vice versa; *N*, number of patient with at least one prescription of the respective drug within 180 days, out of all patients

*p < 0.05, ** *p* < 0.01, ***p < 0.001

^a^Conditional logistic regression models were adjusted for the number of outpatient physician visits per time window and prescription of high-ceiling diuretics (sulfonamides) simultaneously

^b^CLR = odds ratios were retrieved from conditional logistic regression analysis

To evaluate potential confounding by previous hospitalizations, we repeated the primary analysis in a subset of 1444 patients without prior hospitalization. Odds ratios of 2.64 for potassium (95% CI 1.32–5.28, *p* = 0.0062), 2.07 for NSAIDs (95% CI 1.51–2.85, *p* < 0.0001), and 3.37 for amoxicillin/clavulanic acid (95% CI 1.93–5.90, *p* < 0.0001) were slightly but not significantly elevated compared with the total patient population.

Adjustment for visits and prescription of high-ceiling diuretics attenuated the odds ratios for all three drug classes, while the significant positive association remained (Table 2). Subgroup analyses were performed for potentially interacting co-medication. Results are displayed in Table S5 in the supplement.

Treatment with metamizole sodium, paracetamol, and opioids was each significantly associated with hospitalization for heart failure and shock. Our analysis showed a significant positive association with macrolides, but no relevant association with fluoroquinolones (Table 3).

**Table 3:** Comparison to other classes of pain medication and antibiotics

	Windows (days)		Effect	N	Discordant pairs
	Hazard period	Control period	OR_MH_ (95% CI)	With drug (all patients)	Hazard; control
Comparison to other classes of pain medication					
NSAIDs	1–30	31–60	1.80 (1.39–2.33)***	543 (2185)	160; 89
Metamizole sodium					
Patients w/o NSAIDs	1–30	31–60	1.95 (1.35–2.82)***	237 (1642)	84; 43
Patients w/ and w/o NSAIDs	1–30	31–60	2.09 (1.56–2.80)***	370 (2185)	140; 67
Paracetamol					
Patients w/o NSAIDs	1–30	31–60	1.45 (1.15–1.82)**	606 (1642)	175; 121
Patients w/ and w/o NSAIDs	1–30	31–60	1.57 (1.29–1.90)***	884 (2185)	268; 171
Opioids					
Patients w/o NSAIDs	1–30	31–60	1.46 (1.00–2.12)*	228 (1642)	67; 46
Patients w/ and w/o NSAIDs	1–30	31–60	1.87 (1.36–2.55)***	372 (2185)	112; 60
Comparison to other classes of antibiotics					
Amoxicillin/clavulanic acid					
Patients w/o other antibiotics	1–15	16–30	3.60 (2.03–6.38)***	219 (1675)	54; 15
Patients w/ and w/o other antibiotics	1–15	16–30	3.25 (2.06–5.14)***	318 (2185)	78; 24
Macrolides^a^					
Patients w/o amoxicillin/clavulanic acid	1–15	16–30	2.50 (1.10–5.68)*	78 (1867)	20; 8
Patients w/ and w/o amoxicillin/clavulanic acid	1–15	16–30	2.73 (1.37–5.44)**	107 (2185)	30; 11
Fluoroquinolones^b^					
Patients w/o amoxicillin/clavulanic acid	1–15	16–30	0.93 (0.55–1.57)	188 (1867)	27; 29
Patients w/ and w/o amoxicillin/clavulanic acid	1–15	16–30	1.12 (0.70–1.78)	239 (2185)	38; 34

*OR*_*MH*_, Mantel-Haenszel odds ratio; *95% CI*, 95% confidence interval for OR_MH_; *Discordant pairs*, patients exposed in hazard period but not in control period or patients exposed in control period but not in hazard period. *p < 0.05, **p < 0.01, ***p < 0.001

^a^Number of patients with macrolide prescriptions: *n* = 107; number of macrolide prescriptions: *n* = 141; stratified for macrolide: erythromycin: *n* = 4, clarithromycin: *n* = 89; azithromycin: *n* = 48

^b^Number of patients with fluoroquinolone prescriptions: *n* = 239; number of fluoroquinolone prescriptions: *n* = 324; stratified for fluoroquinolones: ofloxacin: *n* = 1; ciprofloxacin: *n* = 216; norfloxacin: *n* = 39; levofloxacin: *n* = 28; moxifloxacin: *n* = 40

## Discussion

Our results confirmed previous studies that identified an increased risk for prescription of NSAIDs and heart failure hospitalization. Additionally, prescription of oral potassium supplements and amoxicillin/clavulanic acid was significantly associated with higher risk for hospitalization.

In a recent review, Greene et al. [35] proposed to intensify the interventions in the outpatient setting if heart failure deteriorates to prevent hospitalization. We found increasing physician contact prior to hospitalization. Increasing frequencies of visits may indicate disease progression and can frame the time period for intensification of patient management. Therefore, evaluating precipitating factors is important. In accordance with the literature, most visits were issued by general practitioners, suggesting that they were primarily treating the HF patients [2].

### Potassium

Potassium supplements are frequently used to balance hypokalemia, which is a common complication in HF patients who are on diuretic treatment [19, 22] and on its own can be a proxy for increased diuretic use. Electrolyte imbalances may be a risk factor for exacerbation of cardiac disorders and potassium supplements can both be interpreted as proxy for hypokalemia and risk factor for hyperkalemia in case of over-use. Our main analysis showed a significant association with heart failure hospitalization. Within 1–30 days prior to index hospitalization, some discordant patients in the hazard period had prescriptions for antibiotics, while no discordant patient in the control period was prescribed antibiotics. We therefore repeated the main analysis in patients without any prescription of an antibiotic during the observation period (*n* = 1456). An odds ratio of 1.8 was found (95% CI 0.96–3.38, *p* = 0.068).

Interestingly, significantly more discordant patients with potassium supplements in the hazard period had closer lab assessments (potassium and renal function) compared with discordant patients in the control period. Closer monitoring itself may indicate a deteriorated general condition. Therefore, risk attributed to potassium supplements may be overestimated.

With respect to the literature, hyperkalemia was reported to increase risk for mortality and readmission in patients hospitalized for HF [21] and was strongly associated with reduced renal function [23, 24]. Additionally, an increased risk for HF hospitalization was reported after an event of hyperkalemia [23].

Co-medication influencing potassium levels may alter the impact of potassium supplements as risk factor [36]: However, numbers of patients with potassium supplements were too small to conclude the effect of ace-inhibitors/sartans. With respect to small sample size, less frequent co-medication such as spironolactone or multiple combinations, e.g., ace-inhibitors with/without concomitant thiazide diuretics, could not be studied in stratified analyses. Propensity-matched cohort studies [19, 22] investigated the association between use of potassium supplements and hospitalization for worsening heart failure on long-term, reporting slightly increased hazard ratios. Potassium supplements were discussed to be markers of disease severity and use of higher doses of diuretics [22]*.* Furosemide and torsemide are commonly prescribed to enforce diuresis in order to prevent heart failure decompensation. Usage indicates a potentially acute condition or deteriorating ejection fraction. We therefore adjusted our analysis for the prescription of high-ceiling diuretics (sulfonamides). Although odds ratios were attenuated, a positive significant association remained. We can conclude that potassium supplement is not solely dependent on high-ceiling diuretics as a risk factor for hospitalization.

### NSAIDs

Prescription of NSAIDs is contraindicated in patients suffering from severe heart failure [37]. In addition, even in the general population, recent NSAID use is associated with increased risk for heart failure hospitalization [6, 7]. Huang et al. [8] conducted a case-crossover study in the National Health Insurance Research Database in Taiwan, evaluating NSAID use in patients without a history of heart failure. They report a 1.58-fold increased risk for a first heart failure hospitalization (adjusted OR, 95% CI 1.40–1.79, hazard period 1–30 days, control period 121–150 days). Although different patient collectives were studied, our results (crude OR 1.8, 95% CI 1.39–2.33, and adjusted OR 1.50 95% CI 1.14–1.97, time period 30 days) are compatible with the overall increase in risk for heart failure hospitalization reported in the literature. Previous studies also found a dose-dependent effect. In addition, varying risks between individual NSAIDs were reported [7, 8, 10, 11].

In our study, only few patients were prescribed coxibs (*n* = 57), which did not allow for risk comparison with respect to cyclooxygenase-2 selectivity. As previously mentioned, we also evaluated paracetamol, metamizole sodium, and opioids to assess potential confounding by pain as underlying condition. We also found an increased risk for hospitalization for these drug classes. Positive associations remained after adjustment (Supplement Table S6). Inhibition of prostaglandin synthesis by NSAIDs is a well-known risk for reduced renal function and increased peripheral resistance [7]. However, the association for the other drugs, which do not share this mechanism, is surprising and likely requires replication. This finding suggests that renal function decrease may not be the only factor contributing to the increased risk by NSAIDs.

Previous studies in current paracetamol users also reported an increased risk for a first-diagnosed episode of heart failure [10] and congestive heart failure [38]. Risk estimates were higher among new users and in patients using high paracetamol doses [10, 38]. Thus, pain itself may be a risk factor for deteriorating heart failure or an early symptom of worsening general condition. We could not consider over-the-counter drugs in this study, which means that every prescription was issued by a physician. It can be assumed that pain leading to physician contact is of higher severity.

### Amoxicillin/clavulanic acid

Prescriptions of amoxicillin and clavulanic acid can serve as proxy for the underlying infection. Infections were reported to be common precipitating factors for HF decompensation requiring hospital admission, especially with respiratory infection being common [12, 13, 15, 16]. Accordingly, our study found a significantly increased risk for hospitalization after amoxicillin/clavulanic acid prescription (OR 3.25, 15 days). Patients with amoxicillin/clavulanic acid were more often co-prescribed for mucolytics or drugs to treat asthma/COPD compared with the whole study population. While an increased risk for severe infections was previously reported for patients suffering from COPD [12], our study found no significant difference in risk for heart failure hospitalization comparing patients with and without drug treatment of asthma/COPD.

In order to evaluate infection as risk factor, we extended the analysis to other classes of antibiotics. Macrolides and fluoroquinolones share a broad spectrum of indications with amoxicillin/clavulanic acid and prescription numbers allowed for their evaluation. We found a positive association for macrolide antibiotics but not for fluoroquinolones. With lacking diagnoses and wide ranges of indications for all three antibiotic classes, reasons remain unclear. Nevertheless, higher rates of respiratory infections in patients receiving amoxicillin/clavulanic acid and macrolides are expected, suggesting some confounding by indication (and its severity): co-prescription with mucolytics was stronger correlated for amoxicillin/clavulanic acid and macrolides (Pearson correlation coefficient 0.25 (95% CI 0.23–0.27, *p* < 0.0001) and 0.18 (95% CI 0.16–0.20, *p* < 0.0001)) than for fluoroquinolones (0.07 (95% CI 0.05–0.09, *p* < 0.0001) within 30 days prior to hospitalization. We also found comparably fewer chest x-rays for fluoroquinolones (amoxicillin/clavulanic acid *n* = 62, 19.5%; macrolides *n* = 18, 16.8% and fluoroquinolones *n* = 26, 10.9%; 30 days). However, assessments of the urinary status was more frequent for fluoroquinolones (fluoroquinolones *n* = 56, 23.4%; amoxicillin/clavulanic acid *n* = 36, 11.3%; macrolides *n* = 11, 10.3%, 30 days), possibly reflecting urinary tract infections as a milder type of bacterial infection in the fluoroquinolone-treated patients.

Postma et al. [39] assessed heart failure events in patients hospitalized for community-acquired pneumonia with respect to antibiotic exposure. Compared with patients on beta-lactam monotherapy, increased hazard ratios were reported for clarithromycin and erythromycin. No increased risk was found for ciprofloxacin, levofloxacin, and moxifloxacin (hazard ratios 0.65 (non-significant), 0.27, and 0.50 (significant)), respectively [39]. Our results suggest that respiratory infection may be a stronger risk factor compared with other types of bacterial infections. Amoxicillin/clavulanic acid is usually well tolerated and may be preferred over other antibiotic classes in elderly and multimorbid patients. However, as clinical differentiation between the diagnoses of heart failure and pneumonia may be difficult, antibiotics may have been prescribed for an exacerbation of heart failure in some cases.

### Limitations

Our study has several limitations: First, Swiss claims data is lacking diagnoses due to legal requirements. We used DRG codes to identify patients with hospitalizations for heart failure and shock. In Switzerland, DRGs reflect the leading diagnosis during hospitalization rather than admission diagnoses. Therefore, development of heart failure during hospitalization cannot be ruled out. No information on heart failure state like the New York Heart Association “NYHA” classification was available. Patients with and without known heart failure prior to hospital admission were both evaluated. Thus, therapeutic treatment and baseline risk for hospitalization might differ. Nevertheless, as each patient served as his/her own controls, differences in patient characteristics were equally distributed over hazard and control groups. NSAIDs and oral potassium supplements are available as over-the-counter drugs in Switzerland. Thus, exposure may not have been captured completely. However, as expenses for these drugs are covered by the health insurance when prescribed by physicians, and physician contact was frequent, it is expected that over-the-counter purchases may occur less frequently in our elderly patient population. Amoxicillin/clavulanic acid and, to some extent, also oral potassium supplements serve as proxies for underlying diseases, namely bacterial infection and hypokalemia. Details on type of infection and potassium levels unfortunately were not available in the dataset. With respect to the case-crossover design, exposure was calculated with the assumption of immediate and actual intake of the prescribed drugs. While NSAIDs are typically used on demand for acute pain relief, some patients may have used them continuously. On demand use may be disassociated from dispensing times and thus carries the risk for exposure misclassification [29]. While the case-crossover design controls for time-invariant confounding, which can be seen as a particular strength, potential progression of existing heart failure or general worsening of the overall health condition in an elderly population introduce confounding [30]. We addressed this issue in several different ways: we restricted the observation period to a maximum of 6 months; we chose the control window for our main analyses to be immediately before the hazard period and adjusted our analyses for physician visits and prescriptions of high-ceiling diuretics. Nevertheless, confounding by disease progression may not have been eliminated completely and result in potential overestimation of risk estimates. Overlapping exposure from the control into the subsequent hazard period, potentially shifting risk estimates towards zero, was addressed by introducing a washout period. Multiple control periods were used simultaneously to account for persistent users, which may have resulted in upward-biased odds ratios [40]. Lastly, the number of patients studied was small, resulting in insufficient statistical power especially when stratification for co-mediation was performed. Thus, conclusions should be drawn with caution and further studies are warranted.

## Conclusion

Our study confirmed the previously reported increased risk for prescription of NSAIDs and heart failure hospitalization. Additionally, prescription of oral potassium supplements and amoxicillin/clavulanic acid was also significantly associated with higher risk for hospitalization. Underlying conditions such as pain, electrolyte imbalances, and infections are likely contributing risk factors. The identification of precipitating factors for heart failure hospitalizations becomes increasingly important in an aging society. This knowledge should be used by physicians to better identify patients at risk and to intensify management early on.

## Electronic supplementary material

ESM 1 (DOCX 139 kb)

## Data Availability

The datasets analyzed during the current study are not publicly available as they are part of the confidential Helsana health insurance claims database. Additional information not included in the paper is available from the corresponding author on reasonable request.
