# Supplementary material for: Medication as a risk factor for hospitalization due to heart failure and shock: a series of case-crossover studies in Swiss claims data
Source: Eur J Clin Pharmacol. 2020 Apr 8;76(7):979–89. doi: 10.1007/s00228-020-02835-x (PMC7306029; doi:10.1007/s00228-020-02835-x)
Supplement: Supplementary file 1 — (DOCX 139 kb) [file 228_2020_2835_MOESM1_ESM.docx]

**Supplement**

**Medication as a Risk Factor for Hospitalization due to Heart Failure and Shock: a Series of Case-Crossover Studies in Swiss Claims Data**

European Journal of Clinical Pharmacology

Annika M. Jödicke, Andrea M. Burden, Urs Zellweger, Ivan T. Tomka, Thomas Neuer, Malgorzata Roos, Gerd A. Kullak-Ublick, Ivanka Curkovic,

Marco Egbring^*^

^*^Corresponding Author:

Dr. med Marco Egbring, MBA HSG

Department of Clinical Pharmacology and Toxicology, University Hospital Zurich, University of Zurich, Zurich, Switzerland

[marco.egbring@usz.ch](mailto:marco.egbring@usz.ch)

| **Table S1: Main ICD-10-codes for SwissDRG Grouping** | |
| --- | --- |
| ICD-10 Code | Main Diagnosis |
| [I11.00](https://manual40.swissdrg.org/icd_codes/54771e6ee4b0887aa13fed3b?locale=de) | Hypertensive heart disease with (congestive) heart failure: Without a record of hypertensive crisis |
| [I11.01](https://manual40.swissdrg.org/icd_codes/54771e6ee4b0887aa13fed3c?locale=de) | Hypertensive heart disease with (congestive) heart failure: With a record of hypertensive crisis |
| [I13.00](https://manual40.swissdrg.org/icd_codes/54771e6ee4b0887aa13fed43?locale=de) | Hypertensive heart and kidney disease with (congestive) heart failure: Without a record of hypertensive crisis |
| [I13.01](https://manual40.swissdrg.org/icd_codes/54771e6ee4b0887aa13fed44?locale=de) | Hypertensive heart and kidney disease with (congestive) heart failure: With a record of hypertensive crisis |
| [I13.20](https://manual40.swissdrg.org/icd_codes/54771e6ee4b0887aa13fed47?locale=de) | Hypertensive heart and kidney disease with (congestive) heart failure and renal insufficiency: Without a record of hypertensive crisis |
| [I13.21](https://manual40.swissdrg.org/icd_codes/54771e6ee4b0887aa13fed48?locale=de) | Hypertensive heart and kidney disease with (congestive) heart failure and renal insufficiency: With a record of hypertensive crisis |
| [I50.00](https://manual40.swissdrg.org/icd_codes/54771e6ee4b0887aa13fedf6?locale=de) | Primary right heart failure |
| [I50.01](https://manual40.swissdrg.org/icd_codes/54771e6ee4b0887aa13fedf7?locale=de) | Secondary right heart failure |
| [I50.11](https://manual40.swissdrg.org/icd_codes/54771e6ee4b0887aa13fedf8?locale=de) | Left ventricular failure: Without discomfort |
| [I50.12](https://manual40.swissdrg.org/icd_codes/54771e6ee4b0887aa13fedf9?locale=de) | Left ventricular failure: With discomfort during heavy physical stress |
| [I50.13](https://manual40.swissdrg.org/icd_codes/54771e6ee4b0887aa13fedfa?locale=de) | Left ventricular failure: With discomfort during lighter physical strain |
| [I50.14](https://manual40.swissdrg.org/icd_codes/54771e6ee4b0887aa13fedfb?locale=de) | Left ventricular failure: With discomfort at rest |
| [I50.19](https://manual40.swissdrg.org/icd_codes/54771e6ee4b0887aa13fedfc?locale=de) | Left ventricular failure, unspecified |
| [I50.9](https://manual40.swissdrg.org/icd_codes/54771e6ee4b0887aa13fedfd?locale=de) | Heart failure, unspecified |
| [J81](https://manual40.swissdrg.org/icd_codes/54771e6ee4b0887aa13fef9a?locale=de) | Pulmonary Edema |
| [R57.0](https://manual40.swissdrg.org/icd_codes/54771e6ee4b0887aa14008ce?locale=de) | Cardiogenic shock |

SwissDRG F62: Heart Failure and Shock

The table indicates the main diagnoses eligible to code DRG ‘F62’. Source (German): [https://manual30.swissdrg.org/drgs/54771e6ce4b0887aa13fd397?locale=de](https://manual40.swissdrg.org/drgs/54771e6ce4b0887aa13fd397?locale=de)

F62A: Heart failure or shock with severe co-morbidities and dialysis, reanimation or additionally complicating diagnosis;

F62B: Heart failure or shock with severe co-morbidities but without dialysis, reanimation or additionally complicating diagnosis;

F62C: Heart failure or shock without severe co-morbidities

Patient Inclusion Process

**Swiss health insurance data**

n(Patients) = 540’931

2014-2015

Excluded

n = 537'192

Patients with hospitalization for ’heart failure or shock’

n = 3’739

Excluded

n = 1’076

Observation time 180-days pre-admission and discharge within study period

n = 2’663

No overlapping other hospitalization prior to index hospitalization*

n = 2’611

Excluded

n = 52

Excluded

n = 426

Inpatient nursing home prior to index date**

n = 2’185

**Included patients**

**n = 2’185**

**Figure S1: Patient inclusion process**

*Initial hospitalization for other reason, with subsequent admission to other hospital for heart failure and shock

**Patients were excluded in case they had a nursing home stay during the observation period due to reimbursement regulations in Switzerland. Nursing home stays may be covered by a lump-sum issued monthly, sometimes including physician care and drugs and sometimes without.

Frequencies of outpatient physician visits

**
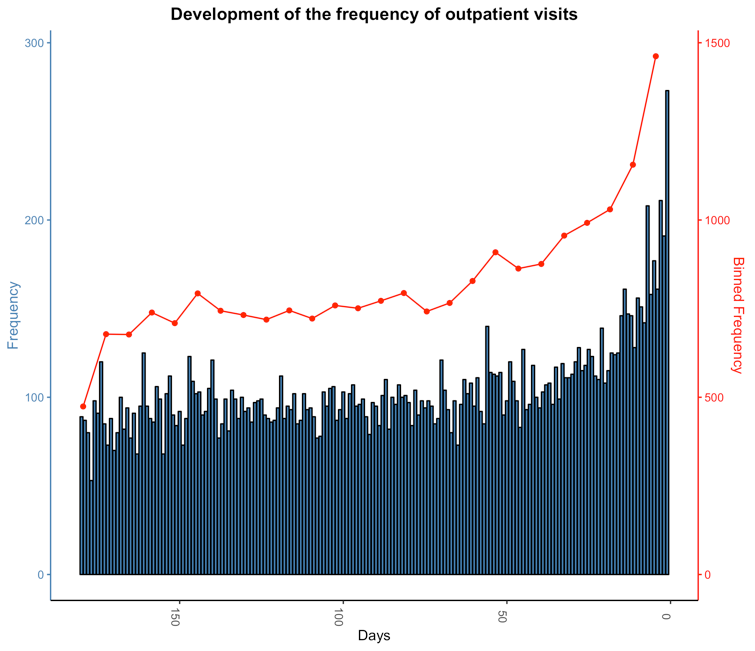
**

**Figure S2: Frequency of outpatient visits within 180 days prior to index hospitalization** Index hospitalization = day 0. The figure indicates the number of individual visits per day issued by all patients for each day towards index hospitalization (blue bars).

To reduce short-term variations visits were binned into 7-day intervals (red line). Based on the binned visits, Spearman correlation coefficient was calculated.

(Spearman **ρ** = 0.56, p=0.0499)

Visits at the index day were excluded.

Study population characteristics: Drug prescriptions

| **Table S2:** Frequent co-medication in all patients (N=2185) | | | | | | | |
| --- | --- | --- | --- | --- | --- | --- | --- |
| **30 days** | | | | **180 days** | | | |
| **ATC** | **N** | **%** | **Name** | **ATC** | **N** | **%** | **Name** |
| C03CA | 666 | 31.2 | High-ceiling diuretics (sulfonamides) | C03CA | 1332 | 62.4 | High-ceiling diuretics (sulfonamides) |
| C07AB | 425 | 19.9 | Beta-blocking agents | C07AB | 1126 | 52.8 | Beta-blocking agents |
| A02BC | 408 | 19.1 | Proton pump inhibitors | A02BC | 1020 | 47.8 | Proton pump inhibitors |
| N02BE | 408 | 19.1 | Paracetamol | B01AC | 927 | 43.4 | Platelet inhibitors |
| B01AC | 316 | 14.8 | Platelet inhibitors | N02BE | 912 | 42.7 | Paracetamol |
| C10AA | 237 | 11.1 | Statins | C10AA | 794 | 37.2 | Statins |
| C09AA | 221 | 10.4 | ACE inhibitors | B01AA | 603 | 28.3 | Vitamin K antagonists |
| N05BA | 215 | 10.1 | Benzodiazepines | C09AA | 591 | 27.7 | ACE inhibitors |
| B01AA | 183 | 8.6 | Vitamin K antagonists | N05BA | 473 | 22.2 | Benzodiazepines |
| N02BB | 180 | 8.4 | Pyrazolones | M02AA | 448 | 21.0 | Antiinflammatory preparations, topical |
| R03AK | 166 | 7.8 | Adrenergics and corticosteroids | C08CA | 444 | 20.8 | Dihydropyridine derivatives |
| N05CF | 149 | 7.0 | Benzodiazepine related drugs | C09CA | 403 | 18.9 | Sartans |
| C01DA | 141 | 6.6 | Organic nitrates | H02AB | 389 | 18.2 | Glucocorticoids |
| H02AB | 141 | 6.6 | Glucocorticoids | A12CC | 371 | 17.4 | Magnesium |
| C08CA | 139 | 6.5 | Dihydropyridine derivatives | N02BB | 370 | 17.3 | Pyrazolones |
| A06AD | 135 | 6.3 | Osmotically acting laxatives | B05BB | 363 | 17.0 | Electrolyte solution |
| A11CC | 134 | 6.3 | Vitamin D and analogues | S01XA | 349 | 16.4 | Other ophthalmologicals |
| B05BB | 127 | 6.0 | Electrolyte solution | A11CC | 347 | 16.3 | Vitamin D and analogues |
| M02AA | 127 | 6.0 | Antiinflammatory preparations, topical | A06AD | 346 | 16.2 | Osmotically acting laxatives |
| B01AF | 126 | 5.9 | Direct factor Xa inhibitors | A12AX | 346 | 16.2 | Calcium, combinations (Vitamin D) |

Top 20 drug classes prescribed to all patients. N = Number of patients with at least one prescription of the respective drug class within 30 or 180 days prior to index hospitalization. ATC = Anatomic Therapeutic Classification

| **Table S3:** Frequent new drug classes prescribed | | | |
| --- | --- | --- | --- |
|  |  | **30 days** | **7 days** |
| **Number of patients with prescription of new drug class** | | 1403 | 630 |
| **Most frequent drug classes prescribed** (top 15) | | N N | |
| **ATC** | **Name** | **30 days** | **7 days** |
| C03CA | High-ceiling diuretics (sulfonamides) | 184 | 76 |
| C07AB | Beta-blocking agents | 130 | 47 |
| N02BE | Paracetamol | 129 | 41 |
| A02BE | Proton pump inhibitors | 112 | 42 |
| N02BB | Pyrazolones | 96 | 32 |
| J01CR | Combination of penicillins, incl. beta-lactamase inhibitors | 88 | 36 |
| B05BB | Electrolyte solution | 82 | 27 |
| B01AA | Vitamin K antagonists | 75 | - |
| C09AA | ACE inhibitors | 71 | 23 |
| A06AD | Osmotically acting laxatives | 70 | 25 |
| B01AC | Platelet inhibitors | 66 | - |
| R05CB | Mucolytics | 66 | 32 |
| J07BB | Influenza vaccines | 63 | - |
| C01DA | Organic nitrates | 62 | 24 |
| C10AA | Statins | 61 | - |
| A03FA | Propulsives | - | 24 |
| C03DA | Aldosterone antagonists | - | 23 |
| J01MA | Fluorquinolones | - | 22 |
| H02AB | Glucocorticoids | - | 20 |
| **Number of patients with prescriptions of new drug class “cardiovascular treatment” (ATC “C”)** | | 555 | 217 |
| **Drug classes further evaluated in case-crossover studies** | |  |  |
| A12BA | Potassium | 33 | 14 |
| M01A | NSAIDs | 104 | 28 |
| J01CR | Combination of penicillins, incl. beta-lactamase inhibitors | 88 | 36 |

New drug classes prescribed within 30 days or 7 days were defined as follows: A patient was prescribed a drug of a drug class that she/he was not being prescribed in the 31-180 days or 8-180 days, respectively. N(Patients) indicated the number of patients that were newly prescribed a drug from the respective new drug class.

Case-crossover studies

| **Table S4: Case-crossover study: Sensitivity analyses – Shorter timeframes** | | | | | |
| --- | --- | --- | --- | --- | --- |
|  | **Windows (days)** | | **Effect** | **N** | **Discordant pairs** |
|  | Hazard period | Control period | cOR**_MH_** (95% CI) | with drug  (all patients) | Hazard; Control |
| **Potassium** |  |  |  |  |  |
| *Main analysis – 20 days* | 1-20 | 21-40 | **2.21 (1.29-3.80) **** | **118** (2185) | 42; 19 |
| *Washout* | 1-20 | 41-60 | **2.00 (1.18-3.38) **** | **118** (2185) | 42; 21 |
| *Multiple control windows* | 1-20 | 21-40, 41-60, […], 161-180 | **3.69 (2.52-5.41) ***** | **118** (2185) | CLR^a^ |
| *Main analysis – 14 days* | 1-14 | 15-28 | **1.65 (0.95-2.88)** | **118** (2185) | 33; 20 |
| *Washout* | 1-14 | 29-42 | **2.13 (1.16-3.94) *** | **118** (2185) | 32; 15 |
| *Multiple control windows* | 1-14 | 15-28, 29-42, […], 155-168 | **3.36 (2.24-5.04) ***** | **118** (2185) | CLR^a^ |
| **NSAIDs** |  |  |  |  |  |
| *Main analysis – 20 days* | 1-20 | 21-40 | **1.87 (1.41-2.47) ***** | **543** (2185) | 140; 75 |
| *Washout* | 1-20 | 41-60 | **2.17 (1.62-2.91) ***** | **543** (2185) | 141; 65 |
| *Multiple control windows* | 1-20 | 21-40, 41-60, […], 161-180 | **1.81 (1.49-2.19) ***** | **543** (2185) | CLR^a^ |
| *Main analysis – 14 days* | 1-14 | 15-28 | **1.33 (0.99-1.79)** | **543** (2185) | 104; 78 |
| *Washout* | 1-14 | 29-42 | **1.90 (1.38-2.61) ***** | **543** (2185) | 110; 58 |
| *Multiple control windows* | 1-14 | 15-28, 29-42, […], 155-168 | **1.79 (1.45-2.21) ***** | **543** (2185) | CLR^a^ |
| **Amoxicillin Clavulanic acid** |  |  |  |  |  |
| *Main analysis – 10 days* | 1-10 | 11-20 | **2.38 (1.51-3.77) ***** | **318** (2185) | 62; 26 |
| *Washout* | 1-10 | 21-30 | **4.40 (2.51-7.71) ***** | **318** (2185) | 66; 15 |
| *Multiple control windows* | 1-10 | 11-20, 21-30, […], 171-180 | **3.82 (2.92-5.01) ***** | **318** (2185) | CLR^a^ |
| *Main analysis – 7 days* | 1-7 | 8-14 | **1.70 (1.06-2.74) *** | **318** (2185) | 46; 27 |
| *Washout* | 1-7 | 15-21 | **2.43 (1.46-4.04) ***** | **318** (2185) | 51, 21 |
| *Multiple control windows* | 1-7 | 8-14, 15-21, […], 169-175 | **3.80 (2.82-5.13) ***** | **318** (2185) | CLR^a^ |

cOR_MH_ = Mantel Haenszel crude odds ratio; 95% CI = 95% confidence interval for OR_MH._ Discordant pairs = patients exposed in hazard period but not in control period or vice versa; N = Number of patient with at least one prescription of the respective drug within 180 days, out of all patients

*p < 0.05,** p < 0.01,*** p <0.001

^a^CLR = Odds ratios were retrieved from conditional logistic regression analysis.

| **Table S5: Case-crossover study: Analysis in subgroups defined by co-medication** | | | | | |
| --- | --- | --- | --- | --- | --- |
|  | **Windows (days)** | | **Effect** | **N** | **Discordant pairs** |
|  | Hazard period | Control period | OR_MH_ (95%CI) | with drug  (with co-medication) | Hazard; Control |
| **Potassium** |  |  |  |  |  |
| Main analysis in all patients | 1-30 | 31-60 | **2.04 (1.24-3.36) **** | 118 (2185) | 47; 23 |
| *Subgroups defined by prescription of co-medication^a^* | | |  |  | |
| High-ceiling diuretics (sulfonamides) | 1-30 | 31-60 | **1.85 (0.94-3.63)** | 64 (904) | 24; 13 |
| No High-ceiling diuretics (sulfonamides) | 1-30 | 31-60 | **3.33 (0.92-12.11)** | 21 (853) | NA |
| ACE-inhibitors/sartans | 1-30 | 31-60 | **2.50 (1.10-5.68) *** | 51 (999) | 20; 8 |
| No ACE-inhibitors/sartans | 1-30 | 31-60 | **1.15 (0.55-2.42)** | 42 (837) | 15; 13 |
| **NSAIDs** |  |  |  |  |  |
| Main analysis in all patients | 1-30 | 31-60 | **1.80 (1.39-2.33) ***** | 543 (2185) | 160; 89 |
| *Subgroups defined by prescription of co-medication^a^* | | |  |  | |
| Diuretics | 1-30 | 31-60 | **2.07 (1.45-2.94) ***** | 309 (1221) | 95; 46 |
| No Diuretics | 1-30 | 31-60 | **1.42 (0.86-2.35)** | 134 (575) | 37; 26 |
| ACE-inhibitors/sartans | 1-30 | 31-60 | **1.71 (1.17-2.51) **** | 260 (999) | 72; 42 |
| No ACE-inhibitors/sartans | 1-30 | 31-60 | **2.28 (1.47-3.52) ***** | 200 (837) | 66; 29 |
| Anticoagulants | 1-30 | 31-60 | **3.82 (1.97-7.42) ***** | 120 (506) | 42; 11 |
| No Anticoagulants | 1-30 | 31-60 | **1.57 (1.14-2.17) **** | 333 (1325) | 96; 61 |
| Platelet-inhibitors | 1-30 | 31-60 | **1.84 (1.19-2.85) **** | 199 (713) | 57; 31 |
| No Platelet-inhibitors | 1-30 | 31-60 | **1.80 (1.27-2.55) **** | 288 (1258) | 88; 49 |
| **Amoxicillin / clavulanic acid** |  |  |  |  |  |
| Main analysis in all patients | 1-15 | 16-30 | **3.25 (2.06-5.14) ***** | 318 (2185) | 78; 24 |
| *Stratification for co-morbidities^b^* |  |  |  |  |  |
| Asthma/COPD | 1-15 | 16-30 | **2.50 (1.20-5.21) *** | 112 (498) | 25; 10 |
| No Asthma/COPD | 1-15 | 16-30 | **3.79 (2.10-6.82) ***** | 206 (1687) | 53; 14 |

OR_MH_ = Mantel Haenszel odds ratio; 95% CI = 95% confidence interval for OR_MH._ Discordant pairs = patients exposed in hazard period but not in control period or vice versa

NA = not available: No Numbers of discordant pairs displayed due to small numbers to prevent patient identification. *p < 0.05,** p < 0.01,*** p <0.001

^a^Co-medication: Patients were required to be exposed to the respective co-medication during the 90 days prior to index hospitalization, with drug exposure in each of the three 30 days time frames. Prescription duration was estimated with the ‘once per day’ assumption. Patients receiving a prescription of the co-medication of interest but do not fulfill the aforementioned criteria were not evaluated. No co-medication indicates that there was no prescription of that drug class within 180 days prior to index hospitalization. Thus, the sum of patients labelled with and without co-medication does not add up to the total number of patients.

^b^Patients were required to have at least one prescription of a drug for obstructive airway disease (ATC: R03) within 180 days prior to hospitalization.

The ‘number of patients with drug’ refers to patients from the subgroup with a prescription of potassium/nsaids/amoxicillin:clavulanic acid within 180 days.

| **Table S6: Adjustment for disease progression for other drug classes** | | | |
| --- | --- | --- | --- |
|  | **Windows (days)** | | **Adjusted** **analysis^a^** |
|  | Hazard period | Control period | OR_MH_ (95%CI) |
| **Paracetamol** | 1-30 | 31-60 | 1.37 (1.12-1.67) ** |
| **Metamizole sodium** | 1-30 | 31-60 | 1.63 (1.20-2.22) ** |
| **Opioids** | 1-30 | 31-60 | 1.42 (1.02-1.97) * |
| **Macrolides** | 1-15 | 16-30 | 1.80 (0.88-3.68) |
| **Fluoroquinolones** | 1-15 | 16-30 | 0.92 (0.57-1.49) |

OR_MH_ = Mantel Haenszel odds ratio; 95% CI = 95% confidence interval for OR_MH_ *p < 0.05,** p < 0.01,*** p <0.001

^a^Conditional logistic regression models were adjusted for the number of outpatient physician visits per time window and

prescription of high-ceiling diuretics (sulfonamides).
